# Nanocarriers: A Reliable Tool for the Delivery of Anticancer Drugs

**DOI:** 10.3390/pharmaceutics14081566

**Published:** 2022-07-28

**Authors:** Hussein Sabit, Mohamed Abdel-Hakeem, Tahsin Shoala, Shaimaa Abdel-Ghany, Mokhtar Mamdouh Abdel-Latif, Jawaher Almulhim, Mohamed Mansy

**Affiliations:** 1 Department of Medical Biotechnology, College of Biotechnology, Misr University for Science and Technology, Giza P.O. Box 77, Egypt; 2 Department of Pharmaceutical Biotechnology, College of Biotechnology, Misr University for Science and Technology, Giza P.O. Box 77, Egypt; mohamed.abdelhakim@must.edu.eg; 3 Department of Environmental Biotechnology, College of Biotechnology, Misr University for Science and Technology, Giza P.O. Box 77, Egypt; tahsen.shoala@must.edu.eg (T.S.); shaimaa.ibrahim@must.edu.eg (S.A.-G.); 4 Department of Maxillofacial Surgery and Diagnostic Sciences, College of Dentistry, Jazan University, Jazan 45142, Saudi Arabia; mokhlatif@jazanu.edu.sa (M.M.A.-L.); mmansy@jazanu.edu.sa (M.M.); 5 Department of Biological Sciences, King Faisal University, Hofuf 31982, Saudi Arabia; almulhim@kfu.edu.sa

**Keywords:** polymer nanocarriers, anticancer, drug delivery, nanomedicine

## Abstract

Nanomedicines have gained popularity due to their potential therapeutic applications, especially cancer treatment. Targeted nanoparticles can deliver drugs directly to cancer cells and enable prolonged drug release, reducing off-target toxicity and increasing therapeutic efficacy. However, translating nanomedicines from preclinical to clinical settings has been difficult. Rapid advancements in nanotechnology promise to enhance cancer therapies. Nanomedicine offers advanced targeting and multifunctionality. Nanoparticles (NPs) have several uses nowadays. They have been studied as drug transporters, tumor gene delivery agents, and imaging contrast agents. Nanomaterials based on organic, inorganic, lipid, or glycan substances and synthetic polymers have been used to enhance cancer therapies. This review focuses on polymeric nanoparticle delivery strategies for anticancer nanomedicines.

## 1. Introduction

Cancer is one of the deadliest diseases in industrialized and developing countries, and researchers are constantly searching for new treatments. Therapeutic options for cancer have risen enormously over time, but we are far from finding a magic bullet to cure it. We may never find a catch-all therapy based on current evidence, so we must keep inventing and finding different approaches to tackle this disease [1]. The growth in cancer incidence and mortality affects conventional cancer care delivery internationally. Integrative oncology provides a platform for exploring and implementing safe, effective, and standard cancer treatment. It can assist in bridging healthcare gaps by offering evidence-informed, patient-centered care [2]. However, cancer is considered a significant global health problem due to death and treatment consequences [3].

Conventional imaging, diagnosis, and treatment continue to fight cancer. These practices’ risks, side effects, and limitations can affect patient results. Therefore, cancer must be viewed, diagnosed, and treated in new, safe, and reliable ways [4].

Several modalities are available to treat cancer. These include, but are not limited to, chemotherapy, radiation, hormonal therapy, palliative therapy, immunotherapy, viral-mediated therapy, and surgery. However, nanotechnology appears to be a reliable approach to tackling most of the problems related to anticancer drug delivery.

Nanotechnology is an area of science dealing with the manufacture of nanosized particles with sizes ranging from 1 to 100 nm via various synthetic processes, particle structures, and particle size changes [5,6,7]. The scientific breakthrough of regulating materials at the nanoscale offers the prospect of a significant shift in therapeutic medical procedures. Nanoparticles are increasingly being used in various areas, including the biological sciences, physics, chemistry, medicine, and material sciences [8]. It has been discovered that shrinking bulk materials to nanosize changes their physicochemical properties, which can be exploited in various biomedical applications. Nanomaterials are especially interesting for a wide range of biomedical applications because of their high surface-to-volume ratio, ability to interface with either molecular or cellular processes, and influence their capabilities. Nanomaterials are especially interesting for a wide range of biomedical applications [9].

The present review describes different approaches to treating cancer, with some emphasis on the nanocarrier-mediated delivery of anticancer drugs.

## 2. Existing Art

Chemotherapy is commonly used, and researchers have discussed ways to overcome acquired drug resistance, which reduces therapy efficacy. One alternative is to treat cancer as a chronic condition instead of eradicating it. In the early 2000s, metronomic therapy (low chemotherapy dosages delivered constantly) targeted endothelial cells and reduced drug resistance [10]. However, the safety, efficacy, and side effects profiles are not clearly understood [11]. Chemotherapy’s effects on numerous organ systems can be challenging to distinguish from malignancy and systemic disorders with immunosuppression [12]. Therefore, searching for new, safe, and effective therapies is in global demand.

Cancer immunotherapy is a well-established and effective strategy for cancer treatment. Given the extensive academic and clinical study efforts devoted to expanding endogenous and synthetic immunotherapy methods, it is critical to focus on critical topics and identify bottlenecks to fundamental knowledge and clinical success [13]. Recent cancer immunotherapy advancements have rekindled optimism for a cure. Immune checkpoint inhibitors are used to treat numerous cancers [14]. Moreover, immunotherapy has improved cancer patients’ clinical outcomes. However, mutations, lack of selectivity, insufficient tumor-reactive T cells, and host immunosuppression might restrict the clinical benefits of immunotherapy [15]. Other different approaches to treating cancer are presented in Table 1.

## 3. Anticancer Drugs

Anticancer medicine, often known as an antineoplastic drug, is any drug that is successful in treating malignant or cancerous illnesses. Anticancer drugs are classified into several types, including alkylating agents, antimetabolites, natural substances, and hormones. Furthermore, there are several medications that do not fall into these categories yet have anticancer activity and are thus used in cancer treatment. Chemotherapy is sometimes mistaken for using anticancer drugs, although it refers to chemical substances used to treat illnesses in general. Mechlorethamine, a nitrogen mustard that was discovered to be effective in treating lymphomas in the 1940s, was one of the first drugs used clinically in modern medicine to treat cancer. In 1956, antimetabolite methotrexate was the first pharmaceutical to treat solid tumors. The following year, 5-fluorouracil was the first of a new family of tumor-fighting treatments, known as first pyrimidine analogs. Since then, several anticancer drugs have been developed and used with remarkable success [37,38].

Cytotoxic medicines, hormones, and signal transduction inhibitors are three types of pharmaceuticals. All alkylating agents, antibiotics, antimetabolites, and other drugs are cytotoxic, which destroys cells, particularly dividing cells. As a result, all of the terminology and broad concepts apply to cytotoxic drugs. Hormonal medications treat malignancies that affect hormone-sensitive organs, such as the breast and prostate. Some drugs do not always fall neatly into these two categories [39].

DNA alkylating medications are still utilized to treat cancer, despite their toxicity. The prevailing belief is that rapidly proliferating tumor cells expose more single-stranded DNA, making them more sensitive to alkylating drugs. As our understanding of DNA repair pathways expands, it becomes clear that defective DNA repair, a hallmark of cancer, plays a role in determining the therapeutic window of these dangerous drugs. Although novel alkylating patterns are unlikely to enter clinical trials, these medications have provided us with better knowledge of the therapeutic potential of targeting the DNA damage repair pathway [40].

Several previously approved drugs for uses other than cancer treatment have recently been revealed to have cytostatic effects on cancer cells. Because these drugs have already been tested for toxicity in humans and animals, they might be easily repurposed as anticancer therapies. Among the recently recognized probable cytostatics are benzimidazole anthelmintics (albendazole, mebendazole, flubendazole), antihypertensive drugs (doxazosin, propranolol), psychopharmaceuticals (chlorpromazine, clomipramine), and anti-diabetic therapy (metformin, pioglitazone) [41].

More anti-tumor medications are being developed because of the increased prevalence of cancer. From 2015 to 2020, fifty-six novel small-molecule anticancer medicines were authorized and classified into ten groups based on their anti-tumor target properties. TKIs (30 medications), MAPK inhibitors (3 drugs), CDK inhibitors (3 drugs), PARP inhibitors (3 pills), PI3K inhibitors (3 drugs), SMO receptor antagonists (2 drugs), AR antagonists (2 drugs), SSTR inhibitors (2 drugs), IDH inhibitors (2 pills), and others are examples of these (6 drugs). PTK inhibitors (30/56) are among them, and they have resulted in a paradigm change in cancer treatment, with reduced toxicity and greater potency [42].

Tyrosine kinases are classified into two types: receptor tyrosine kinases (RTKs) and non-receptor tyrosine kinases (NRTKs) (nRTKs). The former is responsible for extracellular signal transduction into the cell, while the latter is responsible for intracellular communication [43]. The insulin receptor is part of the RTK family, as are receptors for numerous growth factor families, including vascular endothelial growth factor (VEGF), fibroblast growth factor (FGF), platelet-derived growth factor (PDGF), and epidermal growth factor (EGF) [44].

Tyrosine kinase inhibitors (TKIs) can therefore be divided into many types, including anaplastic lymphoma kinase (ALK) [45]. Fms-like tyrosine kinase (FLT3) is a receptor tyrosine kinase that plays a role in the pathogenesis of acute myeloid leukemia (AML) [46,47]. EGF receptors (EGFR) are cell-surface receptors belonging to the ErbB family of tyrosine kinase [48], and vascular endothelial growth factor inhibitors (VEGF) play a critical role in angiogenesis, which promotes cell survival. The growth and proliferation of endothelial cells by binding to specific receptors (VEGFR-1, VEGFR-2, neuropilin) [49], the smoothened (SMO) receptor, a member of the G protein-coupled receptor family, has emerged as an attractive therapeutic target for the treatment and prevention of human cancers [50], fibroblast growth factor receptor inhibitors, and Tropomyosin receptor kinase inhibitors [51].

Similar to the vesicles formed by lipids (liposomes), amphiphilic block copolymers form vesicles containing an aqueous compartment in the core that encapsulate and protect drugs, peptides, proteins, and enzymes. A glucose oxidase loading polymersome-based nanoreactor was constructed by self-assembling PEG-block-phenylboronic ester or piperidine-functionalized methacrylate PEG-b-(P(PBEM-co-PEM)). Such a nanoreactor is inactive in normal tissues, whereas it is activated in the tumor by its acidic pH (~6.4), thereby increasing tumor oxidative stress by producing hydrogen peroxide by the catalysis of glucose oxidase. Meanwhile, increased hydrogen peroxide induces the self-destruction of nanoreactor-releasing quinone methide to deplete glutathione levels, ultimately suppressing the antioxidant capacity of tumor cells. As a result, the nanoreactor efficiently damages cancer cells and ablates tumor growth via the synergistic effect [52].

The mitogen-activated protein kinase cascade (MAPK/ERK pathway) is a signaling system that is triggered in response to diverse stimuli to regulate the proliferation and survival of various eukaryotic cells and malignant cells [53,54].

Cyclin-dependent protein kinases (CDKs) are representative Thr/Ser phosphokinases that play multifaceted roles in vital cellular machinery in organisms [55]. Dysfunctions of these multifunctional CDKs have been proven to contribute to severe diseases, including cancer, Alzheimer’s, Parkinson’s disease, and strokes [56,57].

Phosphatidylinositol 3-kinase (PI3K) is an enzyme that phosphorylates phosphatidylinositol (PI) at its 3-hydroxyl position to produce phosphatidylinositol 30-phosphate [58]. Aberrant activation of the PI3K/AKT/mTOR (Mammalian target of rapamycin) pathway has been associated with many human cancer types [59,60].

The androgen receptor (AR) is a ligand-dependent transcriptional factor and an important therapeutic target for prostate cancer. Competitive binding of antagonists to AR can alleviate the aberrant activation of AR in prostate cancer [61,62]. Chemotherapeutic agents, such as Camptothecin, Methotrexate, Paclitaxel, and DOX, have been coupled to Somatostatin receptor (SSTR) SSTR2-preferential SST analogs, displaying significant SSTR-selective anti-tumor abilities in many different types of tumors [63].

Histone deacetylase (HDAC) enzymes, along with histone acetylase, control changes to core histone acetylation [64]. Increased HDAC levels have been reported in several human tumors and cancer cell lines [65], including the MM. HDAC inhibitor area class of antineoplastic agents targeting the epigenome, specifically chromatin remodeling, resulting in modulation of genes responsible for apoptosis, cell cycle regulation, and hyperacetylation of many non-histone proteins [66].

## 4. Tackling Cancer

One of the most recent approaches to treating cancer is nanomaterials. Over the last decade, it has been documented that these materials offer new ways to deliver drugs to the target site, further increase solubility and bioavailability, and decrease cytotoxicity. Furthermore, nanotechnology for cancer treatment focuses on early tumor detection and diagnosis using nanodevices to target and deliver chemotherapeutic medications to the tumor site. Gold nanoparticles, for example, have long been considered a possible tool for cancer diagnostics and medicine delivery—high surface-to-volume ratio, surface plasmon resonance, surface chemistry and multi-functionalization, simple synthesis, and stability. Gold nanoparticles are non-toxic, non-immunogenic, permeable, and retain medicines at tumor locations. Innovative gold nanoparticle techniques are being developed.

## 5. Nanocarriers

Because they are inert, these nanocarriers have excellent biocompatibility and are frequently regarded as safe mediums. These nanocarriers circulate for an extended period and constantly release medications, bypassing the endosome–lysosome process [67]. Modifying the physiochemical features of nanocarriers, such as their surface, composition, and shape, can increase activity while reducing side effects [68]. As a result, it has far-reaching implications for pharmaceutical distribution. Even though many nanocarriers have been produced, only a handful have demonstrated an exceptional capacity to transport medication to the targeted area. Some of the characterizing features of nanocarriers are their improved biodistribution and pharmacokinetics, increased dissolution, toxicological prevention, and continuous and precise targeted drug delivery [69,70].

## 6. Types of Nanocarriers

Nanocarriers can be divided into three major types: organic nanocarriers, inorganic nanocarriers, and hybrid nanocarriers.

## 7. Organic Nanocarriers

Solid lipid nanocarriers, liposomes, dendrimers, polymeric nanocarriers, micelles, and viral nanocarriers are examples of organic nanocarriers. These organic nanocarriers are very flexible, have few adverse effects, and may be used with a wide range of medications and binders for drug delivery. Because of enhanced penetration and endurance, organic nanocarriers, such as micelles and liposomes, can aggregate at the appropriate location [71]. Because they are simple compounds, liposome-mediated drug delivery and polymeric nanocarriers are first-generation nanocarriers [72].

## 8. Solid Lipid Nanocarriers

Solid lipid nanocarriers have been used as viable carriers for administering lipophilic medicines since the early 1990s. Solid lipid nanocarriers are made by dissolving solid lipids in water and then combining them with emulsifiers via micro-emulsification [73]. Solid lipid nanocarriers are prepared at room temperature using solid lipids, such as free fatty alcohol or acids; steroids or waxes; mono, di, or triglycerides [74]. The drug molecules can be incorporated into the matrices, crust, or center of the solid lipid depending on the production conditions and composition.

This solid lipid nanocarrier has the potential to overcome the limitations of conventional chemotherapy due to its adaptability. When ionic and hydrophilic drug molecules are combined, the Reticule Endothelial System removes the typical solid lipid nanocarrier, challenging sustained drug release. Previously, researchers discovered how to combine ionic and hydrophilic anticancer drugs with lipophilic pharmaceuticals using solid lipid nanocarriers. Polymer–lipid hybrid nanocarriers, for example, have been investigated as a potential method of oral medication administration [75].

To solve the drawbacks of typical solid lipid nanocarriers, certain newly created nanocarriers, such as nanostructured lipid carriers (a blend of liquid and solid lipids) and lipid drug conjugates (a water-insoluble carrier molecule), have been identified. This nanocarrier could be used for medication delivery via topical, injectable, or oral routes. The solid lipid nanocarrier can be used to deliver any therapeutic molecule to a specified place as an excellent tailor-made carrier. Various investigations in solid lipid nanocarriers have been conducted to act as a vehicle for providing specific nucleic acids or genes [76], to cure ocular diseases, for a limited release of bioactive substances [77], and for precisely timed delivery of anticancer medicinal medicines [78].

## 9. Liposome

Liposomes are spherical vesicles generated by lipid bilayers containing an aqueous core that may transport lipophilic and hydrophilic medications to the target location. The bilayer characterizes it as a vesicle with one or two bilayers. This vesicle acts as a transport agent for physiologically active chemicals in the correct place. However, these molecules have a shorter half-life in systemic circulation. Therefore, liposomes can be PEGylated or stealth liposomes by covering them with polymeric substances, such as polyethylene glycol. Because of the Reticule Endothelial System deception, this stealth liposome has high durability and an extended half-life duration in circulation, resulting in prolonged drug release [79]. The incorporation of bioactive substances into liposomes increases their pharmacokinetics and use. Compared to the medication in solution, doxorubicin in stealth liposomes minimizes plasma administration and potency in healthy cells [80]. Temperature responsive liposomes, for example, have been shown to improve medicine delivery [81].

## 10. Dendrimers

Dendrimers are large branching molecules with a central core (activator core) that generates different arms (terminal active groups) [82]. Dendrimers can also be made from nucleotides, sugar molecules, and amino acids. They are multivalent, branching, contain various peripheral groups, and have a specific molecular weight, making them a one-of-a-kind drug delivery vehicle. A unique, well-organized dendrimer branching pattern may be produced via stepwise dendrimer manufacturing [83].

A generation is a branching stage that is integrated into the center and, when stretched, forms a large outside cluster. Hydrophobic bonding, chemical interactions, or hydrogen bonds might all be employed to encapsulate bioactive compounds within core pores, resulting in improved external functioning. Covalent bonds can thus connect medicinal compounds with active groups at the ends of their chains. These dendrimers have a well-defined structure that may be efficiently adjusted to encapsulate various drugs, such as the anti-tuberculosis medication rifampicin [81]. Single-generation dendrimers, on the other hand, can dissociate the molecules to which they are attached [84].

The pattern of physiochemical interaction is the primary mechanism of drug–dendrimer binding. This dendrimer has the potential to be employed in a variety of applications, including magnetic resonance scanning, targeted treatment, pharmaceutical administration, and antiviral and vaccine delivery [85]. It might potentially be used alongside prodrugs. Several anticancer medicines, such as cisplatin and doxorubicin, have been considerably coupled with dendrimer to provide a more significant anticancer impact [86].

## 11. Polymeric Nanocarriers

**Figure 1:**
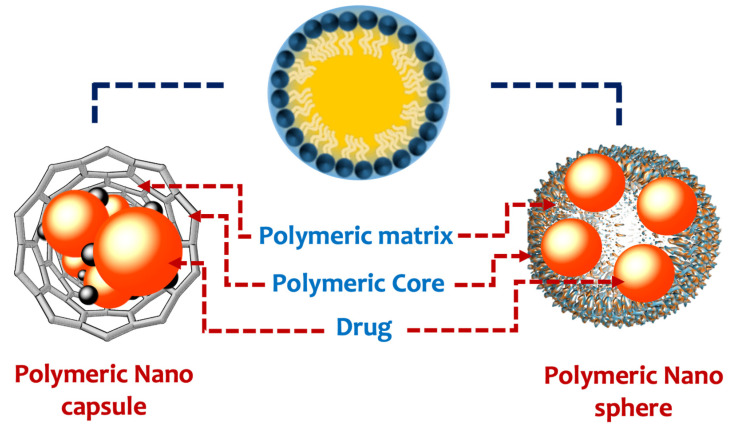
Types of polymeric nanoparticles.

**Figure 2:**
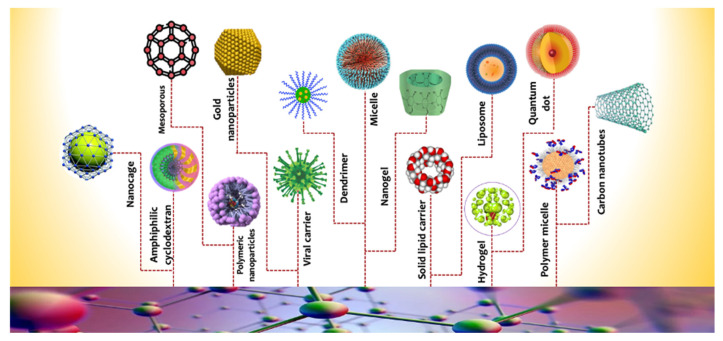
Different types of nanocarriers.

Polymeric nanoparticles are colloidal and stable nanostructures constructed of natural or synthetic polymers [87]. This might be of the storage variety (nanocapsules), which dissolve/disperse bioactive molecules inside the polymer core, or of the substance variety (nanospheres), which entraps bioactive molecules within the polymer matrix (Figure 1). In this context, polymeric nanoparticles can be applied as drug vehicles for cancer treatment [88]. The main types of polymeric nanocarriers are illustrated in Figure 2.

This polymeric nanocarrier outperforms other nanocarriers in terms of consistency, drug payload, half-life time in systemic circulation, and sustained drug delivery [89].

More particular characteristics of polymeric nanoparticles are their excellent synthetic versatility, which allows the researcher to customize them according to the requirements or final aims [90]. Polymeric design could be carried out directly on biopolymers by chemical derivatization to accomplish specific properties.

Another choice is the preparation of synthetic polymers from their corresponding monomers, allowing for a wide range of structures and applications. However, these synthetic materials may be toxic due to the difficulty of eliminating their residuals from the biosystem. Therefore, natural biodegradable polymers, such as chitosan, protamine, gelatin, albumin, and hyaluronic acid, are now being used and are known to reduce toxicity and enhance biocompatibility [91]. Table 2 summarizes the standard drugs and their nanocarriers in various cancers.

Moreover, more improvements may be achieved in producing polymeric nanoparticles for smart delivery by stimulant-responsive polymers (smart polymers) [92]. These advanced polymers release medications in response to internal (low acidity, oxidation, and enzymatic activity) and exterior environmental cues (temperature, light, ultrasound, magnetic and electric field) [90,93]. Despite these obstacles, polymeric nanocarriers may be utilized in drug delivery technology.

**Table 2:** Drugs and their used nanocarriers in different cancers.

Polymer	Drug	Type of Cancer	Experimental Model	Refs.
Chitosan	Quercetin	Colon cancer	In vivo	[94]
Chitosan/protamine	Curcumin and doxorubicin	Breast cancer	In vitro	[95,96]
Albumin	Gemcitabine	Pancreatic cancer	In vitro	[97]
Albumin	Carnosic acid	Pancreatic cancer	In vitro	[98]
Gelatin	Paclitaxel	Colon cancer	In vitro	[99]
poly lactic acid	Cisplatin and Chloroquine	Oral Squamous Cell Carcinoma	In vitro	[100]
Hyaluronic acid (HA)	Paclitaxel	Ovarian Carcinoma	In vitro	[101]
Poly lactide-co-glycolide (PLGA)	doxorubicin	various	In vivo	[102]
polyethyleneimine–Polylactic acid (PEI–PLA)	paclitaxel	lung cancer	In vivo	[103]
Polyethylene glycol (PEG)	Camptothecin (CPT)SN38	breast cancer	In vivo	[104]
PLGA-PEG	Paclitaxel	various	In vivo	[105]

Table 1 illustrates some examples of polymeric nanocarriers as DDS for cancer treatment.

## 12. Micelles

They are amphipathic molecules with a hydrophobic tail that faces the center and a hydrophilic head that links to the solvents on the outside [106]. Furthermore, an amphoteric molecule can form an inverted micelle with the head facing the core and the tail toward the outside in a neutral solution. The size and shape of the created micelle nanoparticles are determined by the solution parameters (temperature, ionic strength, and pH) and the kind of amphiphilic molecule. Micelle production is determined by the critical micellar concentration (surfactant concentration). If the micellar threshold intensity is not attained, appropriate micelle production will not occur. Polymeric micelles are formed in certain solvents by two copolymers that collaborate with amphiphilic molecules.

One copolymer is dissolved by the solvent but not the others. The center is generated by the insoluble copolymer, while the periphery is formed by the soluble copolymer, from which the micellar ensemble is formed [107]. This polymeric micelle can be used in both industrial and non-industrial settings [108]. In the case of hair follicle illnesses, these polymeric micellar nanocarriers precisely guide the pilosebaceous component. Adapalene wrapped in a micellar nanocarrier, for example, boosted its specific efficiency by 4.5-fold and 3.3-fold, respectively, at a modest dosage [109]. Fluorescently labeled aptamers based on anti-human growth factor receptors in pH-responsive micellar nanocarriers may successfully transport nucleic acids to cancer spots [110].

## 13. Inorganic Nanocarriers

Gold, magnetic nanocarriers, quantum dots, and mesoporous silica are inorganic nanocarriers. Inorganic nanocarriers benefit from tradable characteristics. Inorganic nanocarriers can help with biosensing, cell tagging, retargeting, image processing, and detection. These inorganic nanocarriers are also therapeutically effective [111]. Changing the composition or size of inorganic nanocarriers allows for extraordinary magnetic, plasmonic, and optical capabilities. However, using heavy metals as inorganic nanocarriers may have long-term health effects [112].

## 14. Carbon Nanotubes

Carbon nanotubes can have one or many walls. Carbon nanotubes have a wide range of applications in drug administration due to their exceptional properties, such as high aspect ratio, lightweight with high specific surface area, spiky nanostructure creation, and physiochemical, thermodynamic, biomechanical, and electromagnetic abilities [113]. Endocytosis is made possible by needle penetration, which allows it to pass past barriers, such as cell membranes [114]. Bifunctional nanotubes are hydrophilic and may circulate in the bloodstream for an extended period. In contrast, non-functionalized carbon nanotubes are poisonous and insoluble in water. It can target cancer cells due to its structural consistency, flexibility, and surface modification. Bifunctional carbon nanotubes are frequently employed in this concept to contain or link anticancer medicines, such as Paclitaxel [115], Mitomycin C [116], Doxorubicin, Methotrexate [117], and therapeutic compounds. Carbon nanotubes, aside from smart healthcare, are an excellent resource for a wide range of applications due to their inherent features. Graphene is another important carbon-based prospective use that is effective in drug delivery.

## 15. Gold Nanocarriers

Both top-down and bottom-up methods can be used to create gold nanoparticles. The anisotropies of gold nanoparticles include nanostars, nanorods, nanocages, nanoshells, and nanoprisms. Gold nanocarriers’ refractive indices are among the most critical factors attracting them to healthcare. It enables biomolecules, such as enzymes, carbohydrates, fluorophores, peptides, proteins, and DNA, to attach to gold nanoparticles. This allows molecules to travel more effectively throughout the cell, thereby avoiding any impediments that may occur. The primary use of gold nanocarriers is the accurate imaging of tumor cells [118]. In combination with optical coherence tomography agents, the nanoshell can collect three-dimensional pictures of tissues [119]. In addition to computed tomography, positron emission tomography, and computerized tomography analytics, gold nanocarriers have been used [120].

## 16. Magnetic Nanocarriers

The presence of a magnetic core distinguishes magnetic nanocarriers. This magnetic property, together with its changed features, allows it to be used in biosensing applications [121]. Superparamagnetic nanoparticles are more sensitive to magnetic fields than paramagnetic nanoparticles. Because of its magnetic resonance, the polymer-coated superparamagnetic iron oxide nanoparticle has been widely used as a contrast agent in molecular imaging; it also increases cell and particle consent by boosting internalization. Because paramagnetism is affected by the lack of a magnetic field, superparamagnetic iron oxides are also utilized in the passive targeting of cancer cells [122]. Magnetic nanoparticles include hematite, maghemite, nano ferrites, and magnetite. Magnetic nanoparticles have a unique feature that allows them to be used for targeted treatment, gene therapy, and hyperthermia mediators [123,124]. Epirubicin was attempted to bind in the ferrofluid, resulting in drug accumulation at the desired location. However, deep magnetic field penetration is difficult in animal models. As a result, utilizing magnetic nanocarriers on items near the body is disadvantageous [125]. Magnetofection refers to the use of magnetic nanocarriers in gene and antisense treatments [126]. A paclitaxel-encapsulated size-changeable nanocarrier, Trojan Horse, demonstrated improved tumor cell penetration and controlled drug release with increased cytotoxicity.

## 17. Quantum Dot

They are aquatic nanostructures that release energy [127]. The quantum dot diameter influences luminescence in the UV-near IR range, with thinner nanocrystals (2 nm) emitting blue luminescence and bigger quantum dots (5 nm) emitting red fluorescence. This combination of optical and electrical features, longer luminescence, and substantially lower phototability distinguishes it from other organic dyes. As a result, it is capable of imaging cells. In mice, for example, a quantum dot—peptide combination is used for in vivo tumor vascular targeting. To prevent cytotoxicity, a ZnS shell usually surrounds poisonous cadmium in CdSe quantum dots. This boosted the concentration of nanoparticles in the targeted vascular region. These quantum dots were also effective as management and monitoring systems. Surface modification of quantum dots, for example, with encapsulating tumor peptides, successfully attaches to the nucleolin on aberrant cells, increasing cell survival [128]. Furthermore, the combination of quantum dots with RNA interference has been shown to improve gene suppression [129]. Quantum dots are also used as energy transfer quenchers in charge transport operations [130], quantum dot-fluorescence resonance energy transfer systems, chemiluminescence-resonance-energy transfer acceptors [131], and other applications.

## 18. Mesoporous Silica

Mesoporous silica has a massive porous honeycomb structure that allows it to be combined with additional medicinal molecules. It has a wide range of applications in the biomedical field due to its ease of use and availability. It can contain both hydrophilic and hydrophobic medicines, which may then be connected to a binding pocket for targeted treatment [132]. In cancer treatment, mesoporous silica can be employed for both proactive and reactive cancer targeting [133]. Anticancer drugs, such as camptothecin and methotrexate, are efficiently administered when mesoporous silica is used [134].

## 19. Hybrid Nanocarriers

Hybrid nanocarriers are composed of two or more organic and inorganic nanocarriers that can be employed in combination or separately. Hybrid nanocarriers include lipids, ceramic–polymer hybrids, and other hybrid nanocarriers. When two nanoparticles combine, they have a dual nature of both, significantly improving their properties [135]. Organic nanocarriers, such as liposomes, are less stable because of their internal solution permeability.

This allows it to be cleared swiftly from circulation. As a result of increased stability, it is appropriate for medication delivery. The action location, drug type to be attached, physiological obstacles during drug administration, durability, and bioavailability of the nanocarriers all impact the choice of nanocarriers. Several research initiatives are now being carried out on these hybrid nanocarriers. The mesoporous silica nanoscale lipid bilayer hybrid nanocarrier device demonstrated internalized zoledronic acid delivery with a high retention rate in breast cancer [136].

This technique allows for the stimulation of drug release, which prevents the substance from entering the body too quickly. Albumin hybrid nanocapsules have been reported to help encapsulate hydrophilic peptides or other small therapeutic molecules for cancer cell targeting. It enhances cancer cells’ homogeneous dispersion in the microenvironment and reduces cytotoxicity [137]. Similarly, ferritin has been shown to be quite effective in encapsulating medicinal compounds, and drug release stimulation enables prolonged drug release into the target location [138]. Several studies have been carried out to assess small interfering RNA in vivo delivery using core/shell lipid/cholesterol-grafted poly(amidoamine) hybrid nanocarriers (polyethylene glycol-Liposome/small interfering RNA nanoparticles and peptide Histidine-Alanine-Isoleucine-Tyrosine-Proline-Arginine-Histidine (HAIYPRH), known as T7-Liposome/small interfering RNA nanoparticles, which binds to the transferrin receptor, thereby mediating the transport of nanocarriers across the blood-brain barrier. Because of its long durability and high cellular absorption, this method is effective in theragnostic gene silencing applications [139].

## 20. Functionalization of Nanocarriers

Derivatization involves adding a moiety to the outside of a nanocarrier system. Controlling the nanocarrier–biosystem link and targeting capabilities throughout the therapeutic delivery procedure is crucial [140]. Intracellular drug nanoparticle delivery, for example, will have improved load capacity, selective cytotoxicity, and cellular absorption [141]. Many strategies are deliberately used to improve the functioning of surface nanoparticles with a range of ligands, such as small molecules, biomolecules, surfactants, polymers, dendrimers, and so on. The multivalent surface allows for the covalent or non-covalent conjugation of various bioactive chemicals or biomolecules, resulting in target-specific connections and cytocompatibility [142].

## 21. Functionalization of Nanocarriers by Polymers

Polymer coating of nanomaterials is a flexible approach that may give macromolecular characteristics to nanoparticles, allowing them to be target specific. The polymer coating enables passive cancer cell targeting and long-term medicine administration [143]. Polyethylene glycol (PEG), for example, increases nanoparticle permeability and retention, giving them excellent target selectivity [144]. The stealth nanocarriers promote particle immersion into cancer cells and decrease serum protein retention in the circulation [145].

Specific chemicals added to stealthy nanomaterials successfully target cancer cells. A polymer-containing functionalized nanocarrier is a model of a cooperative nanosystem employed in the passive targeting of murine malignant tissue. The unified nanocarrier platform comprises a gold nanowire capped with polyethylene glycol and doxorubicin-loaded liposomes. When the first ingredient, a gold nanorod, is exposed to infrared light, it works as a photocatalytic transmitter, causing selected malignant cells to heat up. This attracts the second component, a cyclic peptide species that binds to the overexpression of the p32 stress-associated protein in tumor cells. This heat treatment substantially decreased the tumor size [146].

## 22. Functionalization by Tagging Ligand Molecules

Because nanocarriers may attach to fluorescently labeled ligands, they can be employed in biosensing and disease diagnosis [147]. Functionalized electrostatic interactions between nanocarriers and fluorescently labeled cells are an effective nanosystem for recognizing metastatic cells, cancer cells, and healthy cells in murine and human cell lines [148]. The sensory properties of green fluorescent protein complexed gold nanocarriers are modulated by ligand headgroup changes and cell–nanoparticle affinity [142].

Cellular absorption and disintegration are essential aspects of drug administration that are influenced by the charge density of bifunctional nanocarriers; for example, negatively charged cells may be quickly taken up by positively charged bifunctional nanocarriers. For example, the guanidine headgroup is in charge of magnetic nanomaterial derivatization. Negatively charged functionality groups on the nanocarrier’s surface (carboxylate on iron oxide nanocarrier) have also been reported to link with cellular membranes via dispersion or pinocytosis. Furthermore, the neutral surface charge of a bifunctional nanocarrier demonstrates a unique cellular interaction [149].

## 23. Functionalization of Nanocarriers by Biomolecules

Biomolecules, such as monoclonal antibodies, oligonucleotides, proteins, and small interfering RNA, can be utilized to functionalize nanoparticles. Because of their capacity to bind precisely to cell surface receptors, these biomolecules attached to nanocarriers can minimize cytotoxicity and permit targeted drug delivery, resulting in a more efficient therapeutic impact [150]. These biomolecules can be covalently attached to the nanocarriers utilizing alkyl thiol groups or glycol spacer molecules. Because it is biocompatible, this bio-inspired nanocarrier technology can be used for biosensing, bioimaging, and targeted medicine delivery [151]. This bio-inspired nanocarrier’s cell-mimicking ability allows for long-term circulation throughout the biosystem. It can avoid the endothelial reticule system. The doxorubicin-loaded erythrocyte bioinspired membrane nanoprobe is being studied as a possible diagnostic and therapeutic tool.

## 24. Drug Loading in Nanocarriers and Release Strategy

Because of the functional groups on its surface, drug loading and drug release are effective. The three basic strategies for effective therapeutic drug loading within the nanocarrier system are covalent bonding conjugation, encapsulation, and electrostatic interaction.

## 25. Covalent Bonding

Drug loading and drug release are both effective due to the presence of functional groups on their surfaces. Covalent bonding conjugation, encapsulation, and electrostatic interaction are the three primary mechanisms for effective therapeutic drug loading within the nanocarrier system. The nanocarrier–drug combination gradually disperses to the cell membrane, permitting for accurate sustained release into the target region. This covalent coupling enables the development of a stable nanocarrier technology for delivering specific medications [152].

## 26. Encapsulation

Encapsulation is another method of incorporating medicinal medications into nanocarrier systems. The concave surface of the nanocarrier allows therapeutic medication encapsulation. Drugs can be encapsulated conveniently within the hollow interiors of polymeric nanocarriers, nanocapsules, dendrimers, and other nanocarriers [153]. The hydrophobic characteristics of the interior chambers allow for the insertion of additional hydrophobic drugs inside the nanocarrier via hydrophobic contact or hydrogen bonding. Physical interactions might also produce encapsulation. Liposomes contain medications for active or passive topical administration. To release the drug, pH-sensitive neutralization or hydrolysis, thiolysis, and thermolysis techniques are utilized [154].

## 27. Electrostatic Interactions

The solubility of hydrophobic drugs is improved by nanocarriers with functional groups, such as carboxyl and amine groups. Drug molecules interact electrostatically with the nanocarrier system due to their high-density functional groups. Electrostatic contact allows certain nonsteroidal anti-inflammatory medications, such as indomethacin, ciprofloxacin, diflunisal, and ibuprofen, to be readily integrated into the nanocarrier [155].

## 28. Active Targeting

The efficient treatment strategy employs a small ligand on the surface of nanocarriers that actively connects to the appropriate receptor, stays in the target location, and can be uptaken by sick cells. This method is commonly used because it has high specificity in bonding the target spot. The attaching ligand must detect protein receptors that are increased in ill cells but not in healthy ones [156]. For example, on the surface of C cancer cells, proteins have increased. This active targeting enables sick cells to absorb more medication intracellularly. Small molecules, lectins, antibodies, and their fragments, lipoproteins, peptides (arginyl glycylaspartic acid), hormones, glycoproteins (transferrin), polysaccharides, low molecular weight vitamins (folic acid), nucleic acids, and growth factors are examples of targeting ligands. Because of their high surface-to-volume ratio, nanocarriers may also be tailored to target a wide range of moieties. Active targeting surpasses passive targeting because it removes the need for off-site medication delivery and can reduce multi-drug tolerance [68]. Internalization or endocytosis with decreased tumor buildup has aroused interest in active targeting in cancer treatment due to multiple ligand–receptor connections. This indicates the presence of receptors present specifically in cancer cells [154]. Receptors include the transferrin receptor in breast cancer cells, the epidermal growth factor receptor, folate receptors in ovarian or lung cancer cells, and aptamers. Folate ligands have the benefit of being smaller (441 kDa) than antibodies (160,000 kDa) [157]. The smaller molecule would be absorbed more quickly by the target location than by the larger one. Endothelial cells in the blood–brain barrier possess transferrin receptors, allowing transferrin ligands as a targeted agent for anticancer medicine administration [158].

Targeting the leaky vasculature of tumor arteries is an alternate method of active targeting mechanisms. This disrupts nutrition and oxygen delivery to the tumor arteries, resulting in tumor cell death [159]. Vascular cell adhesion molecules, vascular endothelial growth factors, v3 integrins, and matrix metalloproteases may be targeted using active vascular targeting [160].

Vascular targeting reduces penetration and drug resilience because endothelial cell indicators are more lasting than tumor cell markers [161]. Another method for circumventing the endothelium barrier is the active targeting of caveolar features in endothelial cells [162].

To summarize, actively targeting nanocarriers provides the benefit of targeting areas across the body, while avoiding the enhanced permeability and retention impact. Therefore, the reduced production costs of dynamically targeted nanocarriers are critical for developing economically advantageous pharmaceuticals for disease treatment. Previously, the suppressor gene RB94 was encapsulated in anti-transferrin receptor adorned cationic liposomes to treat genitourinary cancers using low-cost promising manufacturing scale-up approaches [163].

## 29. Passive Targeting

Increased bioavailability and retention impact are the primary mechanisms for passive targeting. Cancer causes a leaky vasculature with endothelial cell diameters many orders of magnitude (50–70 fold) larger than healthy blood arteries [164]. This induces uneven angiogenesis due to lymphatic drainage and internal vascular development. Nanocarriers with more than 40 kDa molecular weights can extravasate through inflammatory, tumor, or ischemia tissue’s leaky vasculature. Passive targeting of this leaky vasculature may allow the nanocarrier to get through the endothelial barrier via the interstitial space, allowing the drug-associated nanocarrier to aggregate at the illness site. This is known as the improved permeability and retention effect, which Matsumura and Maeda discovered in 1986 [79]. This enhanced bioavailability and retention action lowers the adverse side effects caused by medication buildup at the sick site. The disadvantage of employing low molecular weight drugs to target nanocarriers is that they scatter and re-enter the circulation. The functional group’s charge and the size of the nanocarriers govern their aggregation at the target site. As blood circulation endurance rises, nanocarriers with hydrophilic surfaces and diameters smaller than 200 nm display increased permeability and retention [165]. As a result, targeting tumor cells based on their immunochemical and pathophysiological features compensates for the inadequacies [166]. Even though increased permeability and retention significantly influence the targeting of passive malignant cells, they are not present in all cancer cells. This size-dependent increase in permeability and retention differs by tumor and patient [167]. The high fluid pressure in the interstitial space, relative hypoxia, extracellular matrix complexity, endosomal escape, and issues with tumor infiltration as endothelial gaps change all impact improved permeability and retention [168]. As a result, studies of better bioavailability and retention effects in various tumors are critical for designing nanocarriers with greater effectiveness and target-specific therapeutic advantages. DOXILTM, the first clinically tested passively directed nanocarrier, is doxorubicin in PEGylated liposomes.

## 30. Challenges Ahead

Although noncarriers represent a novel and reliable approach to delivering anticancer drugs, several challenges should be addressed. Non-specific sequestration (internalization and binding to the surface of scavenger cells) of nanocarriers by the reticuloendothelial system (RES), for example, is one of the critical hurdles to the clinical translation of systemically administered nanocarriers because it impedes the delivery of an optimal dose to the target site (disease tissues) and can raise toxicity concerns [52]. To overcome this unintended RES accumulation, various RES blockade strategies have been developed [169]. The rapid elimination of nanoparticles from the bloodstream by the mononuclear phagocyte system limits the activity of many nanoparticle formulations. The transient and partial blocking of the mononuclear phagocyte system may enhance the performance of a wide variety of nanoparticle drugs [170]. Moreover, one of the major critical obstacles facing the systemic administration of nano-based medications is their non-specific clearance by the liver, which might decrease the delivery efficiency of these drugs to the target organ [171].

## 31. Future Directions

Numerous studies and clinical trials employing polymeric nanoparticles in cancer therapy suggest that combining polymeric nanoparticle-based methodologies in cancer therapy will be a unique and future strategy, resulting in greater efficacy and drug targeting with decreased toxicity [88].

The future of anticancer therapy will require combining existing methods. Therefore, it is vital to understand which techniques perform effectively when combined to obtain the most significant anticancer effect. Optimal cancer combination therapy can be devised by comprehending the precise mechanisms by which medications eradicate tumors. Nanoparticles have been the most promising anticancer approach to date [172].

New measures for the construction of composite combination therapy must be developed, necessitating expanded research on the various categories of combination therapies, our awareness of inter-drug connections in some cases, the spatiotemporal release of anticancer agents, imprecise initiation of the innate immune response by such composite fusion nanomedicine, variability of metastatic foci, diversification of organ environment, and provision of bioactive composites [173].

Such therapies must be created by incorporating clinical studies containing a sufficient number of patients with virtually identical biomarker expression and by developing more effective pre-clinical models. Future cancer nanomedicine and nanotechnology combination immunotherapy with mechanistically sensible therapeutic mixtures is intended to boost “multi-targeted therapy” by interfering with chemoresistance and increasing the effect of therapeutic configurations.

Incorporating proteomics data from clinical samples, composite nanotherapy, and immuno-oncology could also develop extraordinarily effective and precise nanomedicine [174].

In addition, in vitro models, and in vivo pharmacokinetic profiles must be enhanced to increase the cancer therapeutic efficacy of innovative medications and new drug delivery systems.

## 32. Conclusions

Nanotechnology has been actively integrated as a drug carrier to treat various cancers. Nanocarriers enable the delivery of chemotherapeutic agents at increased drug content levels to the targeted spots. Several nano-drug delivery systems designed for tumor targeting have been evaluated in clinical trials. Although this considerable research regarding the use of nanocarriers in drug delivery, and the numerous numbers of designed nanocarriers, extensive research should be conducted further to investigate their safety profile and potential side effects.

## Figures and Tables

**Table 1:** Different approaches to the treatment of cancer.

Approach	Refs.
Oncolytic viruses, along with conventional chemo- and radiotherapy	[14]
Cytokine-based therapies are harnessed to enhance the activity or alleviate the immune-related toxicities of other treatments as well as to target early-stage cancers.	[16,17]
Monoclonal antibodies have been used extensively in the treatment of cancer, but their use is still limited by several factors, such as tumor penetration and cost. A number of nanobodies have been developed and evaluated at different stages of clinical trials for cancer treatment.	[18]
Therapeutic targeting of non-coding RNAs (ncRNAs) represents an attractive approach for the treatment of cancer, as well as many other diseases.	[19]
Oncolytic virotherapy is a therapeutic approach that uses replication-competent viruses to kill cancers. It involves using viruses to selectively replicate in cancer cells, leading to direct cell lysis and the induction of an anticancer immune response.	[20]
p53-targeted therapy involves restoring/reactivating wild-type p53 or removing mutant p53.	[21]
Synthetic lethality targets the loss of function of tumor suppressor, and despite their toxicity, DNA repair genes, as well as amplification and/or overexpression of genes that cannot be directly targeted.	[22]
Nanotechnology approaches	[23,24]
G-protein-coupled receptors (GPCRs) are being considered as cancer treatment targets.	[25]
Human papillomavirus (HPV)-related malignancies and tumor microenvironment	[26]
Virotherapy uses live viruses as a cancer treatment. Advances in molecular biology and virology have boosted cancer virotherapy research.	[27]
Clustered regularly interspersed short palindromic repeats (CRISPR/Cas9)	[1]
RNA interference	[28]
Cell-secreted nanovesicles (exosomes)	[29]
Metabolic therapy	[30,31]
Nanotechnology-based techniques to target cancer mitochondria show promise in cancer therapy.	[32]
Bacteria-influenced tumor immune microenvironment	[33]
Photodynamic therapy is a non-invasive, highly selective cancer treatment.	[34]
The anti-angiogenic gene delivery inhibits the new tumor vasculature formation, thereby abolishing the nutrient and oxygen supply to the tumor cells.	[35]
Suicide gene therapy kills the cancer cells by introducing suicide-inducing transgenes encoding enzymes that convert the prodrug into an active drug locally at the tumor site.	[36]

## Data Availability

Not applicable.
